# UV-VIS Curable PEG Hydrogels for Biomedical Applications with Multifunctionality

**DOI:** 10.3390/gels8030164

**Published:** 2022-03-05

**Authors:** Tina Sabel-Grau, Arina Tyushina, Cigdem Babalik, Marga C. Lensen

**Affiliations:** Nanopatterned Biomaterials (Secr. C 1), Department of Chemistry, Technische Universität Berlin, Strasse des 17. Juni 115, 10623 Berlin, Germany; arina1984@web.de (A.T.); cigdem7189@hotmail.de (C.B.)

**Keywords:** hydrogels, photopolymers, volume holography, photo curing, multifunctional biomedical biomaterials, light-responsive materials

## Abstract

Multifunctional biomedical materials capable of integrating optical functions are highly desirable for many applications, such as advanced intra-ocular lens (IOL) implants. Therefore, poly(ethylene glycol)-diacrylate (PEG-DA) hydrogels are used with different photoinitiators (PI). In addition to standard UV PI Irgacure, Erythrosin B and Eosin Y are used as PI with high sensitivity in the optical range of the spectrum. The minimum PI concentrations for producing new hydrogels with PEG-DA and different PIs were determined. Hydrogel films were obtained, which were applicable for light-based patterning and, hence, the functionalization of surface and volume. Cytotoxicity tests confirm cytocompatibility of hydrogels and compositions. Exploiting the correlation of structure and function allows biomedical materials with multifunctionality.

## 1. Introduction

In tissue engineering and medical science, hydrogels are particularly suitable as tissue scaffolds due to their tunable properties including water content, swellability, diffusivity and stiffness [1,2]. In fact, many applications are opened up by the explicit control over molecular structure and mechanical properties, such as elasticity, cross-linking degree or surface morphology [3]. Using light to control properties, hydrogels are well-suited scaffolds for light-responsive functionality [4]. Such stimuli-responsive hydrogel materials can change their mechanical properties upon exposure to light. However, relatively little has been studied with respect to the their optical properties and little attention has been paid to their potential photonic functionalities [5]. Control over optical properties and the resulting integration of optical functionality open up new opportunities for multifunctional biomedical materials such as advanced intraocular lens (IOL) implants.

Cataract, the irreversible turbidity of the natural lens of the eye, is one of the most common causes for global blindness and can only be treated by replacing the clouded lens with an artificial IOL implant. Among the state-of-the-art IOLs are modern foldable hydrogel lenses [6]. Persistent problems with IOLs include postoperative calcification [7] and secondary cataract [8]. The processes underlying such postoperative clouding, emerging in vivo from interaction with the biological environment, are still not well understood.

This is where the idea of volume holographic structuring comes into play. Prospective IOLs, based on multifunctional biomedical material with integrated optical functionality, could fulfill their function—i.e., to focus the light onto the retina—with an optically structured volume [9]. As a result, the shape and surface of the IOL remain free and available for other purposes. Thus, subsequent surface modifications remain optional to achieve specific interactions with the biological environment. In order to make this possible, we propose a strategy to combine the optical structuring of the volume and a specific modification of the surface. Therefore, volume holographic structuring can be applied for the integration of three-dimensional optical structures with specific functionality in terms of diffractive properties.

Volume holography is a very interesting field of application for photo-responsive polymers, where diffractive structures are induced by a spatially modulated holographic exposure [10]. Holographic elements such as diffractive structures can accommodate classical optical functions, while at the same time being extremely flat in shape and low in weight. This gives rise to a great potential for replacing classical refractive optical systems or extending them with new functionalities. The prerequisite in each case is the availability of suitable, photo-patternable materials that can exhibit function through structure. An example of such diffractive structures with classical optical function includes holographic lenses.

Poly(ethylene glycol) (PEG)-based hydrogels are generally promising as tissue-engineering scaffolds due to their biocompatibility and intrinsic resistance to protein adsorption and cell adhesion [11]. Furthermore, poly(ethylene glycol)-diacrylate (PEG-DA) hydrogels can be used as model materials for the generation of internal 3D patterns [12]. Acrylate-terminated PEG macromers undergo rapid polymerization in the presence of photoinitiators that generate radicals when exposed to light [13]. This makes PEG-DA hydrogels interesting as scaffolds into which desired bioactivity can be tailored via light-based patterning [12]. In this context, it was shown that hydrogels can also be structured photolithographically using diffusion processes—which are the basis for volume holography.

Additionally, the micropatterning of PEG-based hydrogels with gold nanoparticles allows for the fabrication of functionalized PEG-based hydrogel films [14,15]. The integration—and holographic assembly—of nanoparticles in turn enables the modification of optical properties on the microscale and nanoscale in the form of holographic nanoparticle-polymer composite gratings [16].

In all this, the type of photoinitiator (PI) used is key for the specific photo-response of a certain material. The properties of the PI has strong influence on holographic grating formation in the respective material [17]. It also influences how well certain conditions are met, such as resistance to humidity [18]. Eosin-Y (EY) and Erythrosin B (EB) are amongst the possible PIs applied for holographic grating formation in an AA/PVA photopolymer [19]. EY is used as a PI due to its excellent spectroscopic properties, which makes it suitable for use with light sources in the visible range and safe for living organisms [20]. EB can only be used for free radical polymerization [19].

## 2. Results and Discussion

### 2.1. Gel Formation

PEG-DA was mixed with PI (Irgacure, EB and EY, respectively). Films were prepared by photopolymerization with 366 nm for 1 h. In terms of optical transparency, mechanical integrity, flexibility, and stability, the new gels compare well with other gels based on PEG-DA [21,22,23].

For PEG-DA with Irgacure, EB and EY as PI, a minimum concentration of PI was needed to make gel. With less PI concentration, no hydrogel was formed. The minimum concentration for the different PIs is shown in Table 1. We found a minimum PI concentration for producing the new hydrogels with PEG-DA and different PIs to be 0.025% for Irgacure, 0.1% for EB and 0.5% for EY, respectively.

### 2.2. UV-Vis Spectra

**Figure 1:**
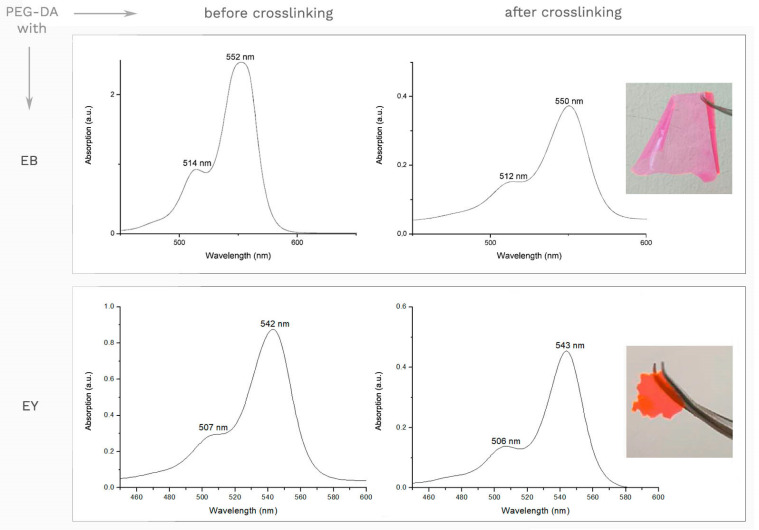
UV-Vis spectra for the novel PIs (EB and EY) with PEG-DA before and after crosslinking, respectively. The hydrogels with new PIs (EB and EY) feature good spectroscopic properties, allowing for use with light sources in the visible range.

Figure 1 shows the UV-Vis spectra before and after crosslinking for the novel PIs. In general, for all new PEG-DA hydrogels, we find spectra shifted somewhat toward higher wavelengths compared to the pure PIs [24,25], while crosslinking tends to cause a small shift toward lower wavelengths, as already substantiated in the literature [26].

While Irgacure, which is an often employed and suitable photoinitiator for biomaterials research, has an absorption maximum in the UV/Vis-spectrum around 300 nm [24], the novel dyes under investigation display a strong absorption of visible light with wavelengths up to 550 nm (see Figure 1).

With EB as PI, the absorption maximum before crosslinking was around 552 nm with **a** shoulder at 514 nm. After crosslinking, the absorption peak can be observed at 550 nm with a shoulder at 512 nm. The typical color for the hydrogel with EB as PI is pink, as shown in Figure 1.

With EY as PI, the absorption maximum before crosslinking was around 542 nm with a shoulder at 507 nm. After crosslinking, the absorption peak can be observed at 543 nm with a shoulder at 506 nm. The typical color for the hydrogel with EY as PI is orange, as shown in Figure 1.

The major advantage of the new PIs (EB and EY) over the standard Irgacure is their high sensitivity in the optical range of the spectrum, which enables optical patterning—e.g., by volume holography. To counter the disadvantage of strong coloring, the composition could be optimized, e.g., by use of a crosslinker so that the concentrations of EB and EY can be reduced, respectively.

In the next step, the respective optical response must be determined depending on the composition. In some cases (such as with an organic cationic ring-opening polymerization system), competing effects regarding the contribution to the optical grating formation can be observed [27]. It is also known that optical shrinkage can have significant influence on grating formation [28] and that the amount of PEG in a composition affects film shrinkage, as well as its optical properties [29]. Furthermore, we have also observed that photoinitiators may contribute to light-induced modification of optical properties and subsequent pattern formation as well [30].

### 2.3. Cytotoxicity Tests

**Figure 2:**
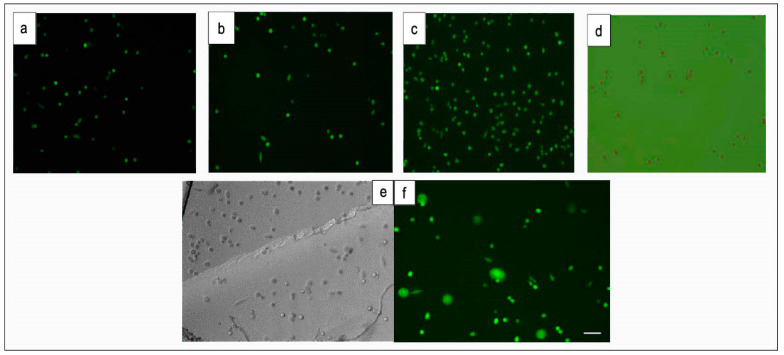
Fluorescent images of cells test by live/dead cell assay with L929 after incubated for 24 h. (**a**) Control cells; (**b**) EB; (**c**) EY; (**d**) EB 0.1% with PEG-DA; (**e**) optical micrograph of cross-linked EB/PEG-DA hydrogel; (**f**) fluorescence image of cross-linked EB/PEG-DA hydrogel. Scale bar depicts 50 µm.

A live/dead staining assay has been used to study the cell viability after incubation with PI EB and EY and also EB 0.1% with PEG-DA before and after crosslinking for 24 h. In the live/dead staining assay, dead cells turn up red, while living cells turn up green when observed with a fluorescence microscope. As shown in Figure 2, all cells but one appear to be green, indicating that PIs and hydrogel (PEG-DA with 0.1% PI EB) are cytocompatible and suitable as substrates for studying the behaviour of L929 cells at the biointerface.

Figure 2 shows fluorescent images of cell tests by live/dead cell assay after being incubated for 24 h with cell line L929. Cell tests shown in Figure 2 confirm cytocompatibility of PEG-DA hydrogels and PIs, hence affirming its aptitude for biomedical applications.

## 3. Conclusions

In addition to the standard photoinitiator Irgacure, Erythrosin B (EB) and Eosin Y (EY) were used as photoinitiators (PI) in PEG-DA hydrogel. We have determined the minimum PI concentration for producing new hydrogels with PEG-DA for the different PIs respectively. All the PIs are cytocompatible and suitable as substrates for studying the behaviour of L929 cells at the biointerface.

The new PIs (EB and EY) feature good spectroscopic properties, allowing for their application with light sources in the visible range and, thus, for applications in volume holography. Cytotoxicity test with cell line L929 were performed to confirm cytocompatibility of hydrogels and PIs. This opens up many options for PEG-DA hydrogels with PIs as multifunctional biomedical applications.

The strategy to combine optical structuring of the volume and specific modification of the surface is particularly interesting for the design of advanced intraocular lens (IOL) implants: based on a multifunctional biomedical material with integrated optical functionality and operating by the principle ‘function by structure’, such a new type of IOL is expected to attain enhanced functionality [9]. The optical functionality of an IOL with integrated holographic lens as a diffractive element consists in focusing the light onto the retina. Several holographic elements can be combined in stacks, where the functionality of the individual elements overlap. The selectivity of a stack then results in a superposition of Bragg selectivity of the individual elements [31].

Beyond an enhanced functionality, the transfer of the optical functionality from the surface into the volume of the IOL implant brings further benefits such as the free interface for specific interaction with the biological environment. As the existing problems with conventional IOLs, such as postoperative clouding, emerge in vivo from interactions with the biological environment, they could be better addressed with free-surface IOLs.

The next step towards such a multifunctional optical material is to better understand the processes that underlay optical structuring, such as the interplay of polymerization and diffusion in the case of holographic gratings. Here, the general approach is to understand holographic grating formation as a consequence of photopolymerization and mass transport processes: local polymerization is induced by a light pattern projected into the photosensitive medium. Polymerization proportional to the light intensity results in the induction of a chemical gradient, followed by monomer diffusion and subsequent polymerization. The final grating is formed as a periodic modulation of optical properties, according to the recording light pattern [10].

It now remains to be clarified what role the individual components play in the formation of optical structures in cases of PEG-DA hydrogels with EB and EY. Furthermore, it remains to be examined if other additives—such as crosslinker or dopant, e.g., in the form of azobenzene-functionalized acrylates or gold nanoparticles—have a positive effect on the formation of optical patterns.

## 4. Materials and Methods

### 4.1. Preparation of PEG Hydrogels

#### 4.1.1. Chemicals

**Figure 3:**
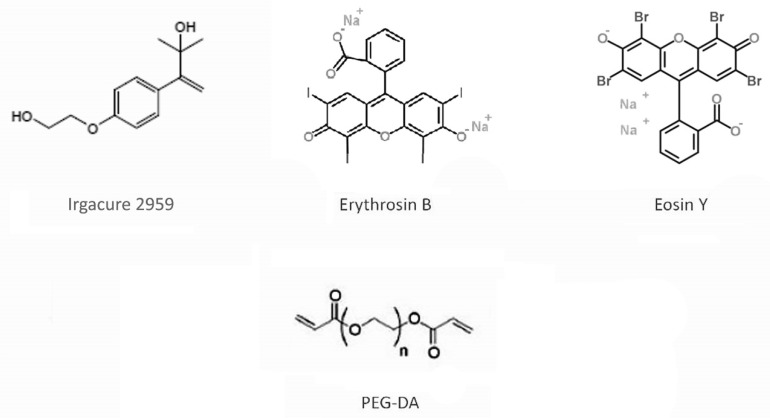
Irgacure 2959, Erythrosin B and Eosin Y are used as photoinitiators (PI); PEG-DA as a precursor for PEG hydrogel.

Poly(ethylene glycol) diacrylate (PEG, Mn 575) and 2-hydroxy-4′-(2-hydroxyethoxy)-2-methylpropiophenone (photoinitiator (PI)—Irgacure 2959), Erythrosin B and Eosin Y were from Sigma-Aldrich Chemie GmbH (Steinheim, Germany). The chemical structures of the hydrogel components are shown in Figure 3.

#### 4.1.2. Synthesis of PEG Based Photopolymer with PIs

PEG-DA was used as precursor. It was mixed with PI (Irgacure, EB and EY, respectively). PI concentration varied between 1% and 6 ppm. The chemical structures of different PIs are shown in Figure 3. For the good mixing of both substances, the mixture was sonicated for around 30 min. At first, the mixture was converted in a cuvette and measured with a UV-Vis spectrometer to obtain spectra before crosslinking. Then, the mixture was dispensed on a glass slide and covered with a thin glass cover slip to achieve a flat and thin hydrogel sample. The glass-sandwich was placed under a UV-light source (366 nm) for 60 min and the glass cover slip was peeled off. A flat, thin standalone hydrogel film was received and also prepared for UV-Vis measurement. Therefore, the hydrogel was placed on a thin glass cover slip and measured with a UV-Vis spectrometer to obtain spectra after crosslinking.

### 4.2. Cell Culture

#### 4.2.1. Chemicals

Mouse fibroblast L929 cells were provided by Dr. Lehmann, Fraunhofer Institute for Cell Therapy and Immunology, IZI, Leipzig, Germany. RPMI 1640 medium, Trypsin, Fetal Bovine Serum (FBS) and Penicillin/Streptomycin (PS) were provided by PAA Laboratories GmbH, Austria, and cell culture plates are from SPL Live Sciences Inc., Seoul, Korea. The Incubator CB150 Series was from Binder GmbH, Germany. Phosphate Buffered Saline solution (Dulbecco’s PBS) was purchased from Sigma-Aldrich Chemie, GmbH, Germany. The counter chamber was from Marienfeld Superior (Paul Marienfeld GmbH & Co., KG, Lauda-Königshofen, Germany).

#### 4.2.2. Cell Culture Experiments

The mouse fibroblasts L929 cells were cultured in the tissue culture plate in RPMI 1640 medium with the addition of 10% Fetal Bovine Serum (FBS) and 1% Penicillin/Streptomycin (PS) in a cell culture plate in an incubator at controlled temperature (37 °C) and CO2 atmosphere (5%). The cells were grown in a cell culture plate and cell culture experiments were performed when a confluency of 75% to 95% was reached.

The hydrogel samples were prior washed with water and kept in a PBS solution for around 30 min before cell culture experiments. As soon as a confluency of at least 75% was reached, the cells were washed with PBS, detached by using trypsin and, after the centrifugation process, a new medium was added on the cells and mixed properly. An amount of 10 μL of this cell medium solution was placed on a cell counter chamber in order to count the cell number by using an optical microscope and to achieve a concentration of 40,000 cells/mL. Depending on the counted cell number, the cell solution was mixed with a defined amount of new medium. The samples were placed in a TCPS plate or on the washed and precut hydrogel. The samples were then cultured within these cells for 24 h, at 37 °C in a 5% CO_2_ atmosphere in a TCPS.

#### 4.2.3. Live Dead Cytotoxicity Assay

The live dead cytotoxicity assay is a fluorescence-based method for checking the viability of cells. Hereby, the cells are stained with fluoresceindiacetete (FBS) and propidiumiodid (PI) molecules. FBS is dissociated in the cytoplasma of live cells into green fluorescence molecules and, due to the size and charge of the PI molecules, they only can enter into the cell cytoplasm when the cell membranes are damaged and are bound to nucleic acids, which appear then as red fluorescence color. In this manner, the live cells appear as green-stained cells and dead cells appear as red-stained cells in a fluorescent microscope image.

For the live dead assay, a 1:1 *v*/*v* solution of PI and FBS in PBS was prepared and added into the cell culture solution in a dark environment. Immediately after the mixtures are prepared, fluorescence images are taken.

### 4.3. Analytical Instruments

UV-Vis spectra were obtained with Cary 4000 UV-Vis Spectrometer (Agilent Technologies, Santa Clara, CA, USA). The spectral range from 300 to 900 nm was measured at room temperature. Liquid samples (before crosslinking) were converted in a cuvette for measurement. The flat, thin standalone hydrogel films (after crosslinking) were placed on a thin glass cover slip for measurement with a UV-Vis spectrometer.

The results from cell culture experiments were observed via the optical microscope from Carl Zeiss, Germany, and analyses were performed with the AxioVision V4.8.2 software (Carl Zeiss, Oberkochen, Germany).

## Figures and Tables

**Table 1:** Minimum PI concentration to make gel for the different PIs.

Photoinitiator (PI)	Minimum PI Concentration to Make Gel with PEG-DA
Irgacure 2959	0.025%
EB	0.1%
EY	0.5%

## Data Availability

Not applicable.
